# Thawed cryopreserved synovial mesenchymal stem cells show comparable effects to cultured cells in the inhibition of osteoarthritis progression in rats

**DOI:** 10.1038/s41598-021-89239-8

**Published:** 2021-05-06

**Authors:** Kiyotaka Horiuchi, Nobutake Ozeki, Kentaro Endo, Mitsuru Mizuno, Hisako Katano, Masako Akiyama, Kunikazu Tsuji, Hideyuki Koga, Ichiro Sekiya

**Affiliations:** 1 Center for Stem Cell and Regenerative Medicine, Tokyo Medical and Dental University, 1-5-45 Yushima, Bunkyo-ku, Tokyo, 113-8510 Japan; 2 Research Administration Division, Tokyo Medical and Dental University, Tokyo, Japan; 3 Department of Cartilage Regeneration, Tokyo Medical and Dental University, Tokyo, Japan; 4 Department of Joint Surgery and Sports Medicine, Tokyo Medical and Dental University, Tokyo, Japan

**Keywords:** Mesenchymal stem cells, Regeneration

## Abstract

Intra-articular injections of mesenchymal stem cells (MSCs) can inhibit the progression of osteoarthritis (OA). Previous reports have used cultured MSCs, but the ability to use thawed cryopreserved MSC stocks would be highly advantageous. Our purpose was to elucidate whether thawed cryopreserved MSCs show comparable inhibitory effects on OA progression in rats to those obtained with cultured MSCs. Cultured rat synovial MSCs or thawed MSCs were compared for in vitro viability and properties. The inhibitory effect of thawed MSCs on OA progression was evaluated by injecting cryopreservation fluid and thawed MSCs in meniscectomized rats. Cartilage degeneration was assessed using gross finding and histological scores. Cultured MSCs were then injected into one knee and thawed MSCs into the contralateral knee of the same individual to compare their effects. Cultured MSCs and MSCs thawed after cryopreservation had comparable in vitro colony formation and chondrogenic potentials. In the rat OA model, the gross finding and histological scores were significantly lower in the thawed MSC group than in the cryopreservation fluid group at 8 weeks. Finally, cartilage degeneration did not differ significantly after injection of cultured and thawed MSCs. In conclusion, thawed MSCs showed comparable inhibitory effects on OA progression to cultured MSCs.

## Introduction

Osteoarthritis (OA) is the most common joint disease in the world^1^ and a leading cause of disability worldwide. Knee OA is the largest cause of locomotion disorders in older adults^2^, and the prevalence of this disease is anticipated to increase in the coming decades^3^. Currently, no consensus exists regarding disease-modifying therapies that address structural abnormalities of the knee or other joints affected by OA.

Mesenchymal stem cells (MSCs) are a promising source for cell therapies. In recent years, clinical reports on the effectiveness of intra-articular injections of MSCs as a treatment for knee OA are increasing. A systematic review showed that intra-articular injections of MSCs improved knee pain but did not fully demonstrate that the injections inhibited the progression of OA^4^. Many clinical case studies have used cultured MSCs^5–7^, but some studies have used MSCs thawed after cryopreservation^8^. The use of MSCs immediately after thawing is more practical because the timing of the injection can be easily adjusted.

A growing number of papers have compared cultured and thawed MSCs for their properties as MSCs and their therapeutic effects on some diseases**.** While several in vitro studies have shown that thawed MSCs were inferior to cultured MSCs in terms of viability^9^, metabolic activity^10^, proliferation^11^ and differentiation potential^10,12^, other studies have found no differences^13–17^. No consensus has yet been reached in in vitro studies, but some in vivo studies have reported similar immunosuppressive effects between cultured and thawed MSCs^17–19^. However, similar results for disease models including OA are lacking, and an inhibitory effect of thawed MSCs on OA progression has not been described to date, though injections of cultured MSCs have been shown to inhibit the progression of osteoarthritis (OA) in animal studies^20,21^.

The primary purpose of the present study was to elucidate whether thawed MSCs would have a comparable inhibitory effect on OA progression to that seen with cultured MSCs. We first investigated the effects of cryopreservation on the viability and properties of rat synovial MSCs. We then validated the inhibitory effect of cultured versus thawed MSCs on OA progression in a meniscectomized rat model. These results established the primary outcome and sample size for the final experiments, in which we injected cultured and thawed MSCs into the opposite knees of the same individuals to directly compare their effects.

## Results

### Effects of 7-day cryopreservation on the viability and properties of MSCs in vitro

**Figure 1:**
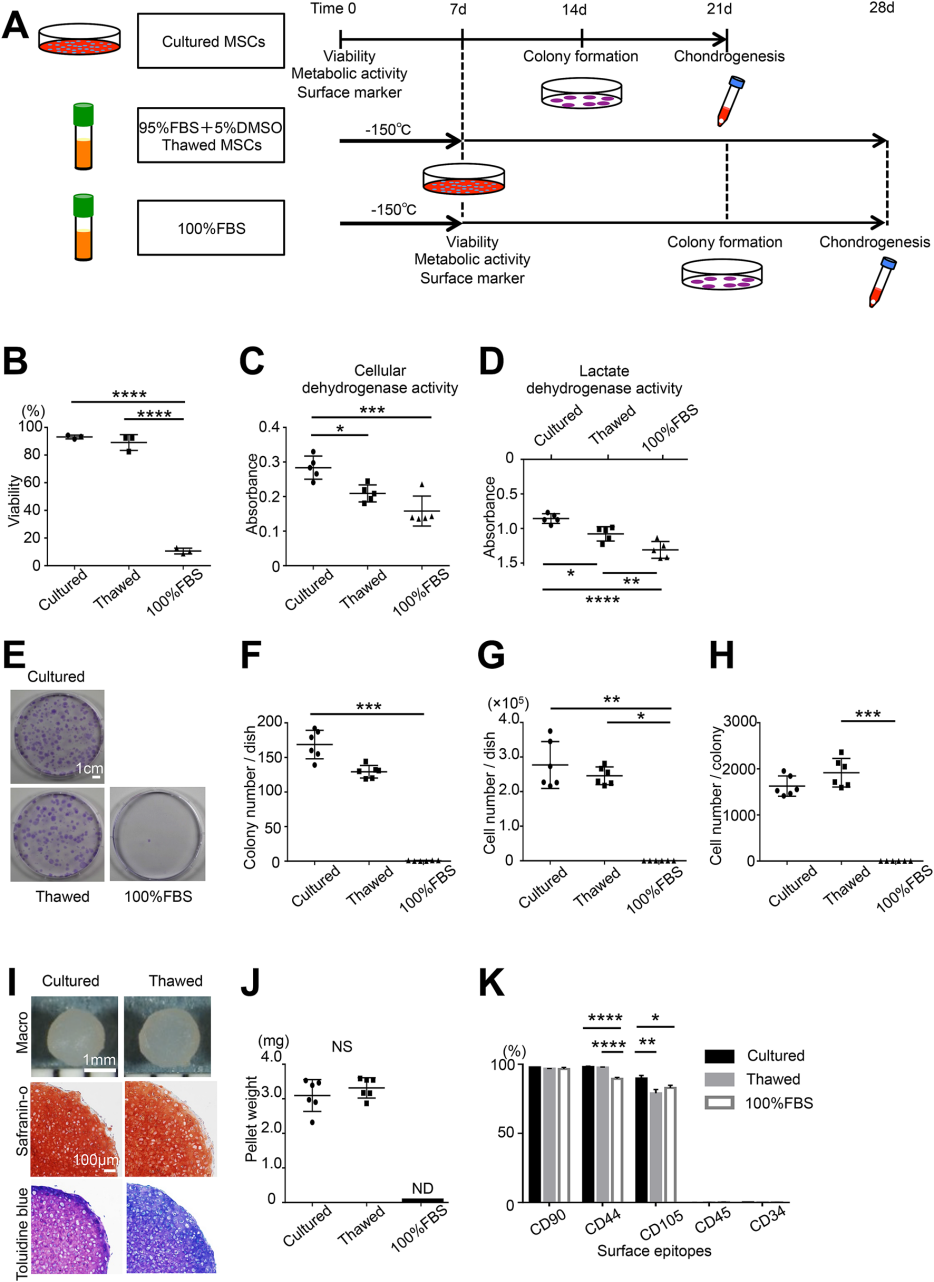
Effects of cryopreservation on the in vitro viability and properties of rat synovial MSCs. (**A**) Scheme: 1 × 10^6^ synovial MSCs were suspended in 1000 μL PBS as cultured MSCs. A sample of 1 × 10^6^ synovial MSCs suspended in 1000 μL preservation fluid containing 95% FBS with 5% DMSO or 100% FBS was cryopreserved at − 150 °C for 7 days for use as thawed MSCs or negative controls. The cells were analyzed for viability, metabolic activity, and surface markers. A 0.5 μL volume of cell suspension (containing 500 cells, including living and dead cells) was allocated to a 60 cm^2^ dish and cultured for colony formation. A 250 μL volume of cell suspension (containing 2.5 × 10^5^ cells, including living and dead cells) was allocated to a 15 mL tube and cultured for chondrogenesis. (**B**) Viability assessed by trypan blue staining. The average with SD is shown (n = 3). (**C**) Cellular dehydrogenase activity was used to confirm live cell metabolic activity (n = 5). (**D**) Lactate dehydrogenase activity was used as a marker of dead cells (n = 5). (**E**) Colony formation: colonies were stained with crystal violet. (**F**) Colony number per dish (n = 6). (**G**) Cell number per dish (n = 6). (**H**) Cell number per colony (n = 6). (**I**) Cartilage pellets and histological images. (**J**) Cartilage pellet weight (n = 6). (**K**) Surface epitopes (n = 3). ND, not detected; *NS* not significant; *p < 0.05, **p < 0.01, ***p < 0.001, ****p < 0.0001 by repeated measures one-way ANOVA followed by Tukey’s multiple comparisons (**B**–**D**,**K**), Kruskal–Wallis test followed by Dunn’s multiple comparisons (**F**–**H**) or Student’s t-test between two unpaired groups (**J**).

Synovial MSCs thawed after cryopreservation in 95% FBS with 5% DMSO or 100% FBS were compared with MSCs without cryopreservation (Fig. 1A). In vitro viability was not significantly different in MSCs thawed after cryopreservation in 95% FBS with 5% DMSO versus cultured MSCs (Fig. 1B), but cellular dehydrogenase activity and lactate dehydrogenase activity were significantly lower in the thawed MSCs as well as in MSCs thawed after cryopreservation in 100% FBS (Fig. 1C,D). Colony formation of MSCs at passage 3 (Fig. 1E), determined as colony number per dish, cell number per dish, and cell number per colony, were not significantly different in thawed MSCs versus cultured MSCs (Fig. 1F–H). MSCs thawed after cryopreservation in 100% FBS did not form cartilage pellets, whereas MSCs thawed after cryopreservation in 95% FBS with 5% DMSO formed similar cartilage pellets to those formed by cultured MSCs (Fig. 1I), and no significant difference was found for cartilage pellet weight (Fig. 1J). Surface antigens in all three groups were consistent with the MSC pattern (Fig. 1K), but CD44 was significantly lower in MSCs thawed after cryopreservation in 100% FBS, and CD105 was significantly higher in cultured MSCs. Representative histograms of surface antigens are shown in Supplementary Figure S1.

### Effects of 16-month cryopreservation on the viability and properties of MSCs in vitro

Synovial MSCs thawed after a 16-month cryopreservation period in 95% FBS with 5% DMSO (thawed MSCs) were compared with MSCs subjected to the same 16-month cryopreservation but cultured for 7 days after thawing (cultured MSCs) (Supplementary Figure S2A). The in vitro viability and cellular dehydrogenase activity were significantly lower in the thawed MSCs compared to the cultured MSCs (Supplementary Figure S2B,C), but the lactate dehydrogenase activity was not significantly different between the thawed MSCs and the cultured MSCs (Supplementary Figure S2D). Colony formation by the MSCs at passage 3 (Supplementary Figure S2E), determined as colony numbers per dish, cell numbers per dish, and cell numbers per colony, were not significantly different between the thawed MSCs and the cultured MSCs (Supplementary Figure S2F–H). The thawed MSCs formed smaller (Supplementary Figure S2I), and significantly lighter weight cartilage pellets compared to the cultured MSCs (Supplementary Figure S2J).

### Cell activity evaluated by luminescence intensity

**Figure 2:**
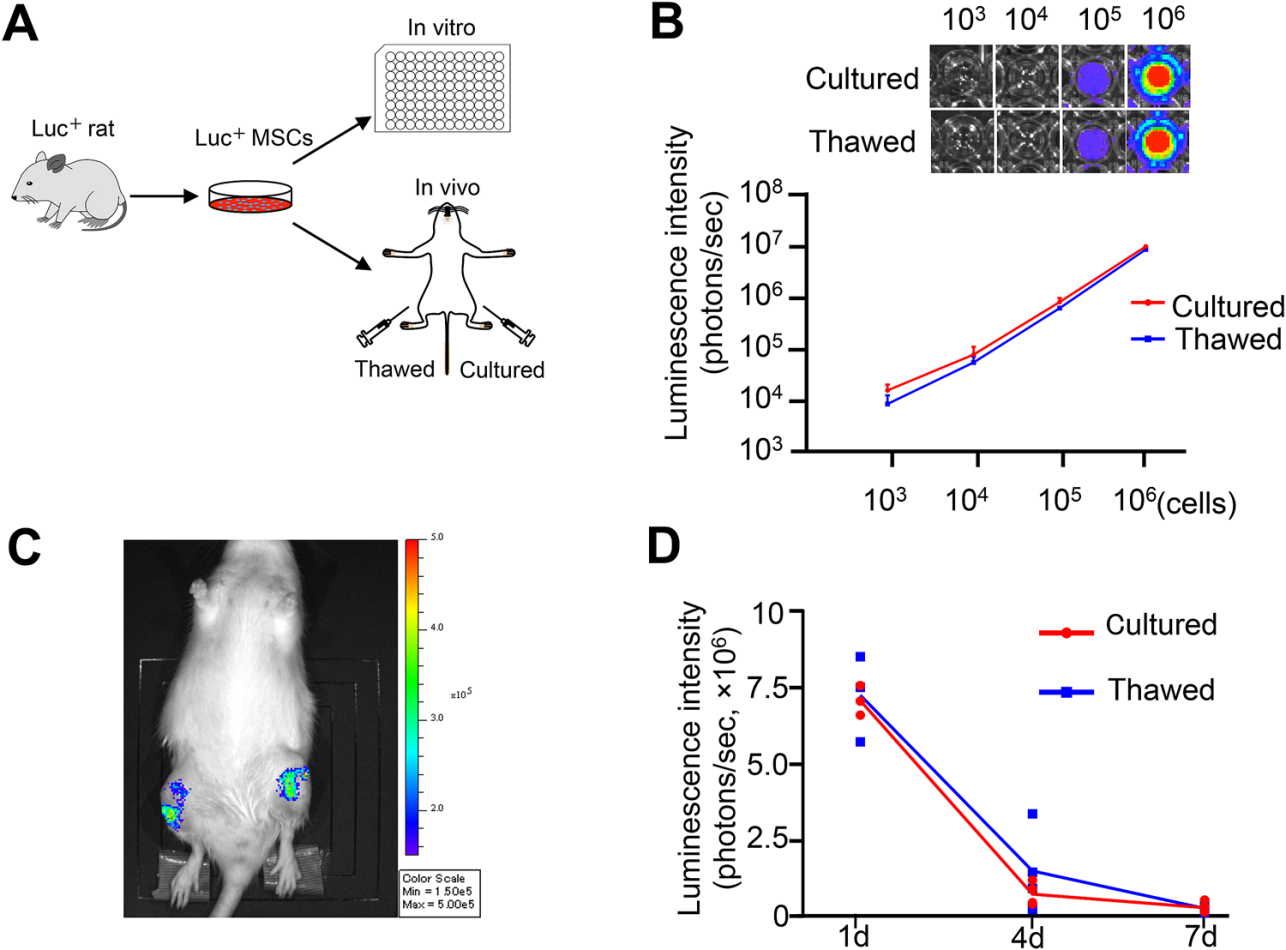
In vitro and in vivo bioluminescence imaging analysis. (**A**) Scheme: synovial MSCs were derived from luciferase-expressing transgenic rats. Cultured MSCs and thawed croypreserved MSCs were plated into 96-well plates and then used for injection into the knees. (**B**) In vitro luminescence images and intensity. Synovial MSCs derived from luciferase-expressing transgenic rat MSCs were allocated into samples of 10^3^, 10^4^, 10^5^, and 10^6^ cells and assessed. The average with SD is shown (n = 3). (**C**) In vivo bioluminescence imaging analysis. The anterior half of the MM in both knees of each rat was removed, 10^6^ cultured MSCs were injected into one knee and 10^6^ thawed cryopreserved MSCs were injected into the contralateral knee without compensation for viability. The scheme and representative images at 1 day are shown. (**D**) Luminescence intensity. The average is shown as a line graph. 1 day (n = 3), and 4 and 7 days (n = 4).

The activity of cultured and thawed MSCs was compared by the luminescence intensity of MSCs expressing luciferase (Fig. 2A). The luminescence intensity in both groups increased as the number of cells increased, but no difference was detected between the groups (Fig. 2B). The two groups of MSCs were then injected into the knees of rats in the OA model and their cellular activities in vivo were compared (Fig. 2C). The luminescence intensity decreased similarly in both groups after 4 days and further decreased after 7 days, with no difference between the two groups (Fig. 2D).

### Inhibitory effect of cultured versus thawed MSCs on OA progression in rats

**Figure 3:**
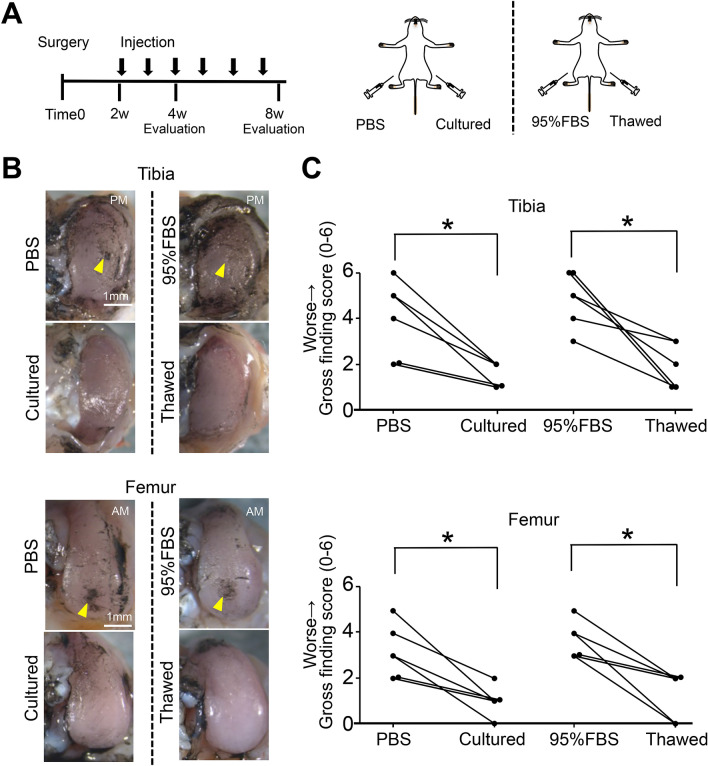
Macroscopic analysis of the inhibitory effect of cultured MSCs versus thawed cryopreserved MSCs on osteoarthritis (OA) progression in rats. (**A**) Scheme. For the rat OA model, PBS was injected into the knee and 10^6^ cultured MSCs suspended in PBS were injected into contralateral knee every week from 2 weeks. Also, 95% FBS with 5% DMSO was injected into the knee and 10^6^ thawed MSCs suspended in 95% FBS with 5% DMSO without compensation with viability were injected into contralateral knee every week from 2 weeks. The knee cartilage was assessed to compare the left and right sides of the same individual at 4 weeks (Supplementary Figs. S1, S2) and 8 weeks. (**B**) Representative macroscopic images for medial tibial and femoral condyles stained with India ink. The images in cultured and thawed groups are inverted horizontally for ease of comparison. Yellow arrow head indicates cartilage erosion. PM, posteromedial; AM, anteromedial. (**C**) Gross finding score. *p < 0.05 by Wilcoxon's signed rank test (n = 6).

**Figure 4:**
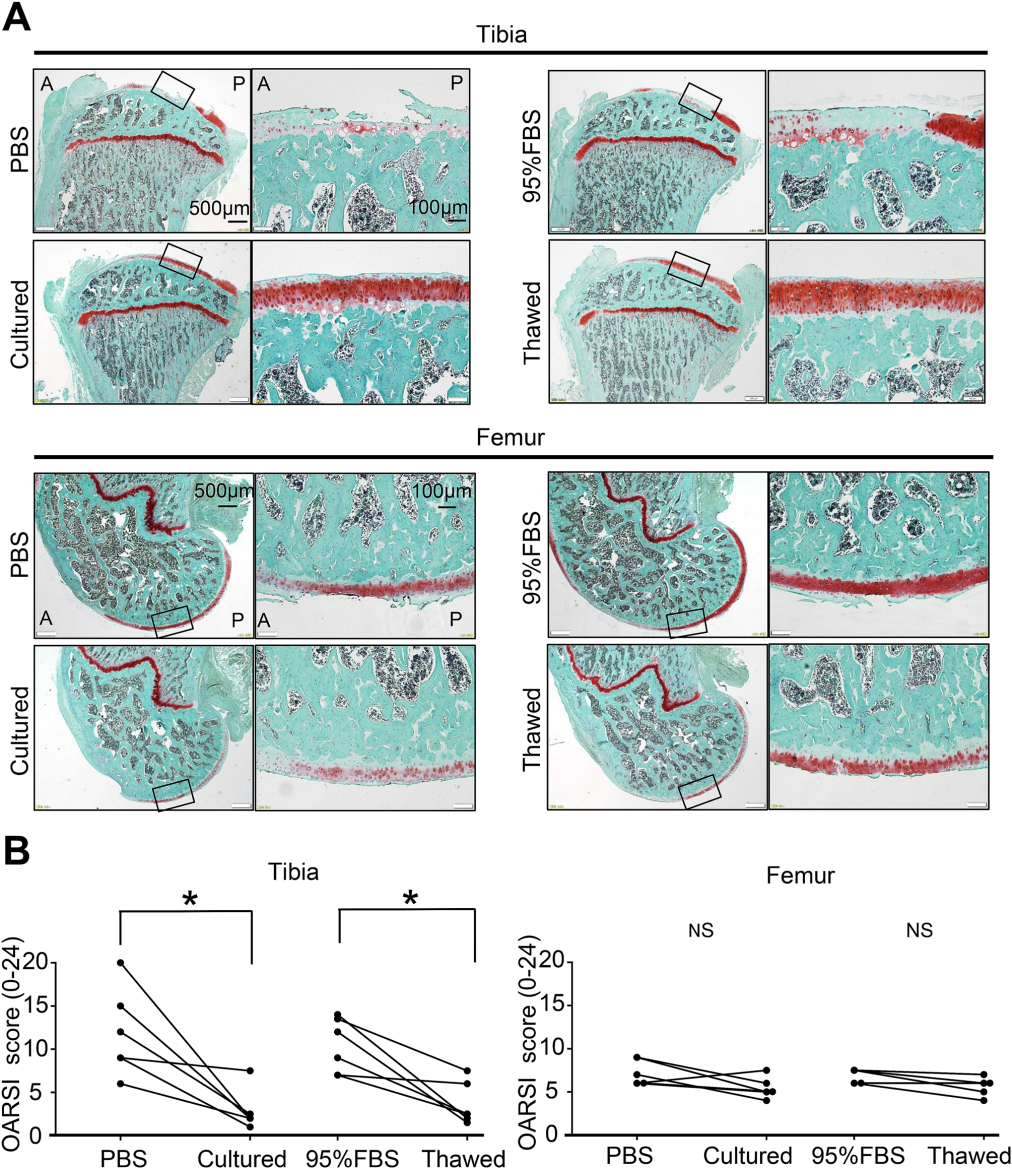
Histological analysis of the inhibitory effect of cultured MSCs versus thawed cryopreserved MSCs on osteoarthritis (OA) progression. (**A**) Representative histological sections stained with safranin O. A, anterior; P, posterior. (**B**) Osteoarthritis Research Society International (OARSI) histological score. NS, not significant; *p < 0.05 by Wilcoxon's signed rank test (n = 6).

PBS was injected into one knee and cultured MSCs were injected into the contralateral knee in one group of OA model rats. In another group, 95% FBS with 5% DMSO was injected into one knee and thawed MSCs were injected into the contralateral knee. The knee cartilage was then assessed to compare the left and right sides of the same individual (Fig. 3A). No macroscopic or histological differences were noted between the PBS and cultured MSC knees or between the 95% FBS and thawed MSC knees at 4 weeks (Supplementary Figures S1, S2). At 8 weeks, macroscopic observation showed erosion of the tibial and femoral cartilage in the PBS and 95% FBS knees (Fig. 3B). The gross finding score for both the tibial and femoral cartilage was significantly higher in the PBS knees than in the cultured MSC knees (Fig. 3C). The gross finding score in both the tibial and femoral cartilage was also significantly higher in the 95% FBS knees than in the thawed MSC knees. Histological observation revealed a decrease in staining of cartilage matrix in tibial cartilage in the PBS and 95% FBS knees at 8 weeks (Fig. 4A). The OARSI histological score in tibial cartilage was significantly higher in the PBS knees than in the cultured MSC knees and was also significantly higher in the 95% FBS knees than in the thawed MSC knees (Fig. 4B). The femoral cartilage did not show as obvious an effect of the two MSC groups as observed for the tibial cartilage.

### Comparison of the inhibitory effect of cultured versus thawed MSCs on OA progression

**Figure 5:**
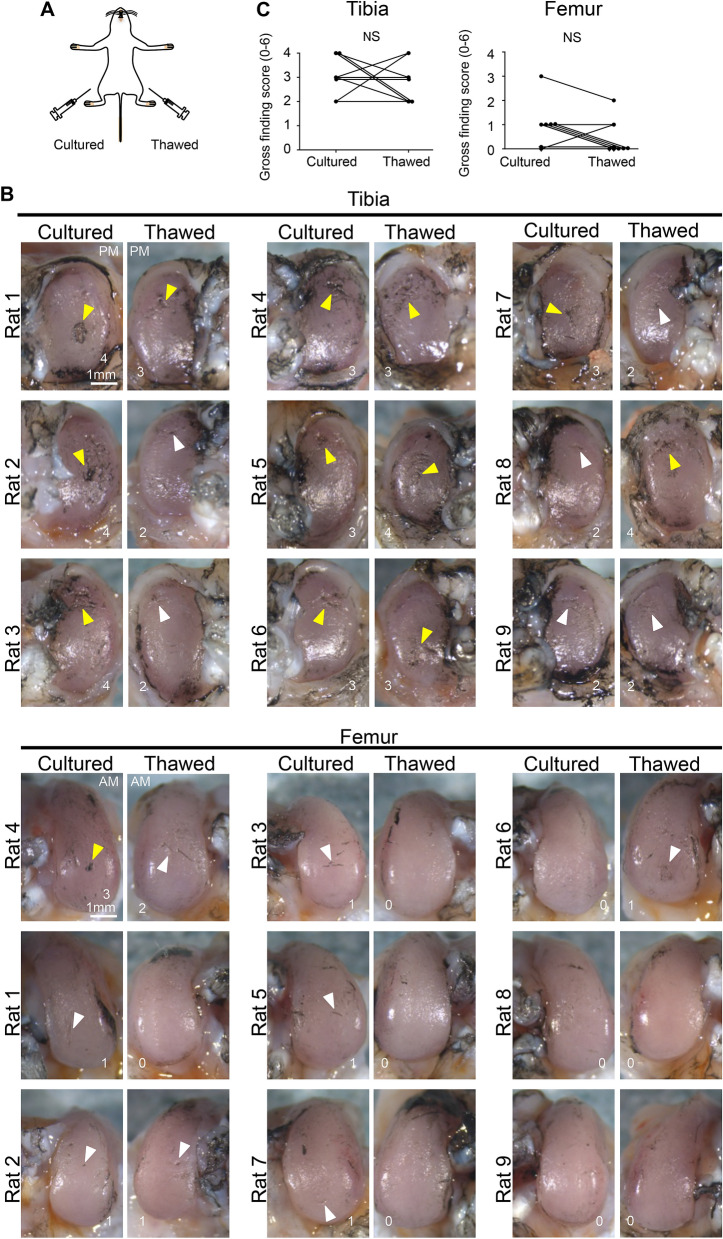
Macroscopic comparison of the inhibitory effects of cultured MSCs versus thawed cryopreserved MSCs on OA progression in both knees of the same rat. (**A**) Scheme: rat OA models were treated with cultured MSCs and thawed cryopreserved MSCs. Cultured MSCs were injected into one knee and thawed MSCs were injected into the contralateral knee every week 2 weeks after the surgery. The knee cartilage was assessed by comparing the left and right knees of the same rat at 8 weeks. (**B**) Macroscopic images of the medial tibial and femoral condyles stained with India ink. All images are shown with gross finding scores. White arrowhead indicates fibrillation and yellow arrowhead indicates cartilage erosion. The images in the cultured cell and thawed cell groups are inverted horizontally for ease of comparison. Images are arranged in the order of the worst gross finding scores on the cultured side. PM, posteromedial; AM, anteromedial. (**C**) Gross finding score. NS, not significant by Wilcoxon's signed rank test (n = 9).

**Figure 6:**
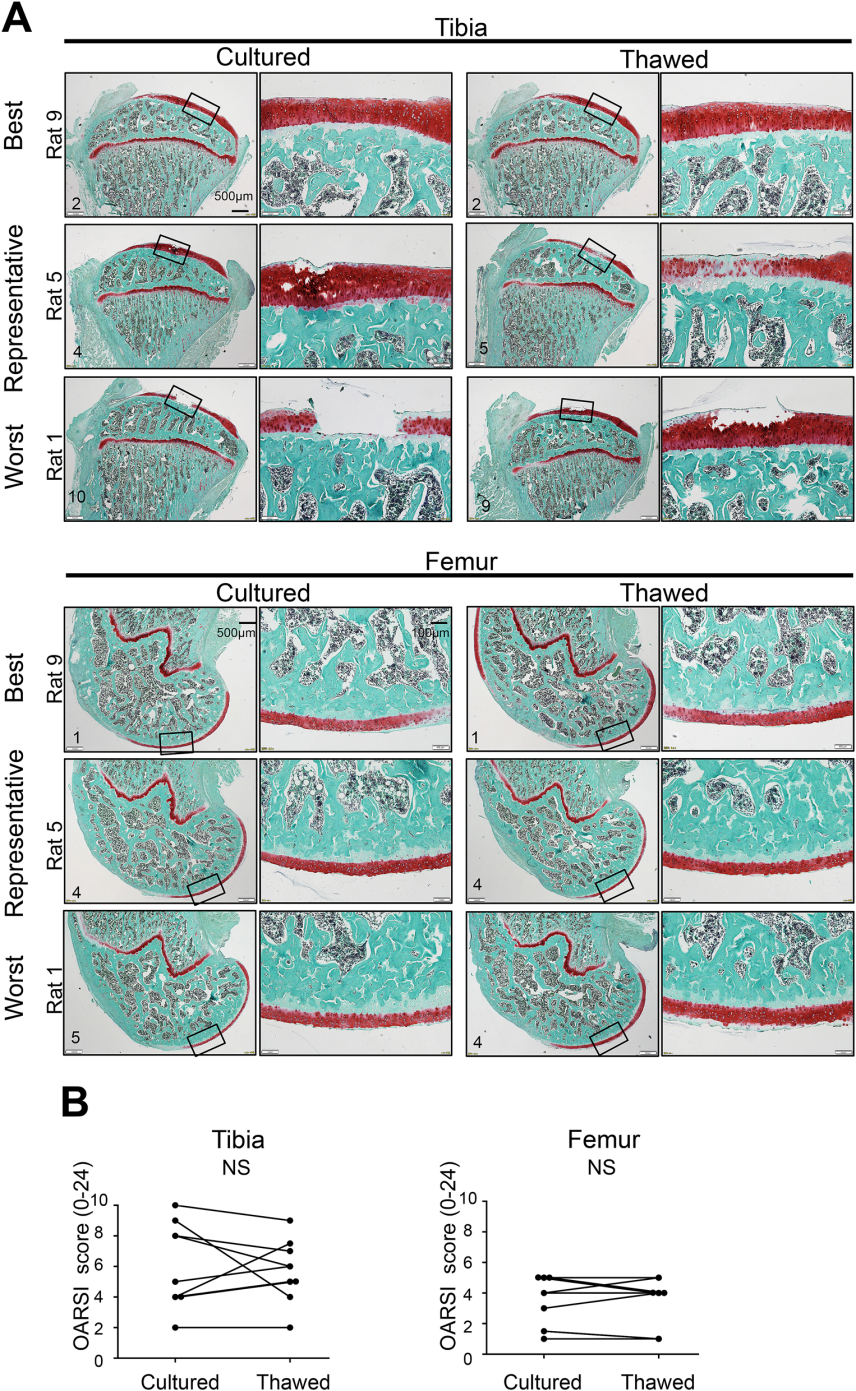
Histological comparison of the inhibitory effects of cultured MSCs versus thawed cryopreserved MSCs on OA progression in both knees of the same rat. (**A**) Histological images for medial tibial and femoral condyles stained with safranin O. Best, representative, and worst images are selected based on Osteoarthritis Research Society International (OARSI) histological scores on the cultured cell side. (**B**) Statistical analysis of OARSI histological score. NS, not significant by Wilcoxon's signed rank test (n = 9).

We calculated the sample size for comparison between thawed MSCs and cultured MSCs using the OARSI histological score for tibial cartilage. Assuming a standard deviation of 2.5 and a correlation between the groups of 0.5, a sample size of 8 was deemed sufficient to detect a difference of 3 calculated by 30% average difference of the OARSI histological score between the PBS and cultured MSC knees (Fig. 4B) with 80% power and 5% level of significance (two-tailed Wilcoxon singed rank test). Considering the dropout rate, we used 9 rats for the experiment. We injected cultured MSCs into the left and right sides of the knee and thawed MSCs into the contralateral knee in nine rats in the OA model and evaluated them at 8 weeks (Fig. 5A). Fibrillation and erosion were seen macroscopically in the tibial cartilage of medial compartments on both sides, while little erosion was seen on both sides in the femoral cartilage (Fig. 5B). No significant difference was observed in the gross finding score for both the tibial and femoral cartilage between the knees treated with cultured or thawed MSCs (Fig. 5C). Histological evaluation of the tibial cartilage showed individual differences from the preserved cartilage matrix to the focal defect in each animal (Fig. 6A). The difference in OARSI histological score between MSCs was 0, − 1.5 to 1 (median, interquartile range) in the tibial cartilage, with no statistical significance (Fig. 6B). The femoral cartilage was less degenerated than the tibial cartilage and no significant difference was detected between the two groups.

## Discussion

We examined the use of 100% FBS, as well as 95% FBS plus 5% DMSO, as cryopreservation solutions in in vitro experiments. The reason for testing 100% FBS was that we expected that it could serve as a negative control for the cryopreservation solution. We have previously reported that 95% FBS plus 5% DMSO was effective, whereas 100% FBS was not effective, for the cryopreservation of human MSCs^22^. The present study confirmed similar results for rat MSCs.

The thawed MSCs and cultured MSCs showed similar luminescence intensity in cells expressing luciferase. The luminescence intensity reflects the activity of the whole cell population^23^. In addition, no difference was found between the two groups following injection into the knees of OA model rats, as the luminescence intensity decreased in a similar time-dependent manner. These results indicated that the activity of the total cell population injected into the joint decreased similarly with time for both cultured MSCs and thawed MSCs.

The thawed MSCs cryopreserved for 16 months exhibited 7.8% lower viability and 22.4% lighter cartilage pellet weight than the cryopreserved but then cultured MSCs, while maintaining their colony-forming ability. The duration of cryopreservation can affect the viability and cartilage pellet weight of the thawed MSCs, since no differences in viability or pellet weight were evident between thawed MSCs and cultured MSCs cryopreserved for 7 days. Carvalho et al. previously showed that thawed rat bone marrow MSCs cryopreserved for 1 month had a lower viability than cultured ones before cryopreservation^24^. Conversely, comparable chondrogenic differentiation abilities were reported in thawed human bone marrow MSCs cryopreserved for 3 weeks by Matsumura et al.^15^ and for 24 months by Mamidi et al.^16^ compared to MSCs cultured before cryopreservation. The difference between our results and theirs could be attributed to the cryopreservation medium, MSC source, MSC species, and analysis method.

Injection of cultured MSCs inhibited OA progression compared to injection with PBS alone. This result is comparable to previous results reported for the rat ACL resection model of OA^20^. Injection of thawed MSCs inhibited OA progression compared to injection with 95% FBS with 5% DMSO. In the control group, the gross finding score varied from 2 to 6 points and the histological score varied from 6 to 20 points, mainly due to the individual differences among the rats. We then compared thawed MSCs and cultured MSCs in both knees to remove individual differences. We set the primary endpoint as the histological score of the tibia, which had the largest difference in scores between the PBS and the cultured MSC knees. The power analysis showed a minimum sample size of 9.

The pooled SD and the correlation were close to the assumed value, so the expected power was maintained. No significant difference was observed in the histological score of the tibia between the knees treated with cultured or thawed MSCs. The gross finding scores of the tibia and femur were also similar. From these results, we concluded that thawed MSCs showed a comparable inhibitory effect on OA progression in rats to that observed with cultured cells. This is the first report showing an inhibitory effect of thawed synovial MSCs on OA progression. In clinical situations, weekly injections of fresh cultured MSCs need repeated cell culture and associated manpower and cost. The ability to use stocks of thawed cryopreserved MSCs would solve these problems and make weekly injections far more convenient for OA patients.

Histological evaluation of the cartilage in the medial compartment showed a more obvious degeneration on the tibial side than on the femoral side in the current OA model. We have previously reported several studies in this rat model of OA, and histological evaluation of the medial compartment cartilage was performed only on the tibia^25–27^. This is because the changes were more marked in the tibial cartilage than in the femoral cartilage. We had resected the anterior half of the medial meniscus (MM) in rats to induce OA. The location of the meniscus defect is constant for the tibial cartilage, whereas it changes for the femoral cartilage with flexion and extension of the knee joint. For this reason, the tibial cartilage is viewed as more affected than the femoral cartilage in this model. Compared to the medial compartment, the cartilage in the lateral compartment degenerated less, but some showed fibrillation upon macroscopic evaluation.

We have shown that weekly injections of MSCs inhibited OA progression in a rat model with an anterior half meniscectomy. We have also previously shown that weekly injections of MSCs suppressed OA progression in a rat anterior cruciate ligament transection model. The effect of weekly injections of MSCs in other OA models will depend on the severity of the OA in the models. For example, in a rat excessive running model^28^, weekly injections of MSCs are likely to inhibit OA progression since this is a mild OA model.

We used 10-week-old female rats in the present study, recognizing that donor factors, such as age and gender, may alter the inhibitory effects on OA. Asumda et al. reported an inferior in vitro chondrogenic differentiation ability of bone marrow MSCs in rats at 15 months of age than at 4 months of age^29^. By contrast, Mochizuki et al. reported a comparable in vitro chondrogenic differentiation potential of human synovial MSCs in 20-year-olds and 70-year-olds^30^. Regarding gender differences, Matsumoto et al. reported a higher chondrogenic differentiation of mouse muscle MSCs in vitro and a greater regeneration of articular cartilage in vivo in males than in females^31^. At present, no consensus has been reached regarding the influence of age; consequently, reports on gender differences are still limited regarding chondrogenic differentiation in MSCs.

Our previous species-specific microarray and PCR analysis of rat synovium after injection of human synovial MSCs showed that most of the synovial MSCs injected into the knee joint migrated into the synovium and expressed PRG-4^32^ and BMPs^33^ for cartilage homeostasis, as well as TSG-6^34^ for anti-inflammation. However, reports on the influence of cryopreservation of MSCs on gene and protein expression are limited. Fu et al. showed that cryopreservation of human umbilical cord-derived MSCs affected the expressions of proteins related to metabolism and cell cycle pathways^35^. We need to clarify whether cryopreservation of synovial MSCs affects the expression of proteins related to cell survival, anti-inflammation, and chondrogenesis.

We performed weekly injection of thawed MSCs in 50 μL 95% FBS, but the use of FBS as a vehicle may complicate the interpretation of our results. FBS contains bovine proteins and could induce sensitization and cause inflammation^36^. Although we did not specifically examine our rats for synovitis, no macroscopic or histological differences were apparent in the tibial and femoral cartilage between the PBS and 95% FBS groups, suggesting that FBS had not significantly induced inflammation in this study. Had we used rat autologous serum instead of FBS, we could have resolved this issue of xenogeneic antigens.

Rat synovial MSCs cryopreserved in 95% FBS with 5% DMSO maintained their viability, colony formation, and chondrogenic abilities, whereas cells cryopreserved in 100% FBS showed obviously reduced viability. Similar results have been reported previously for human synovial MSCs^22^. DMSO in the cryopreservation fluid substantially increased the viability of synovial MSCs. A systematic review of 41 in vitro studies of bone marrow MSCs by Bahsoun et al. showed that cryopreservation does not affect the morphology, expression of surface markers, differentiation, or proliferative potential, but the effects on viability and colony-forming capacity remain to be determined^37^. Variations in viability were mainly related to differences in the cryopreservation solutions, including the DMSO concentrations, and differences in methods used to measure viability.

DMSO is frequently used as a cryoprotectant, but its toxicity can be a problem in clinical applications. Yellowlees et al. reported that intravenous administration 100 g of 20% DMSO for 3 days for the treatment of arthritis caused serious adverse reactions, including oliguria, hemolysis, tremor, and loss of consciousness in one of two elderly patients^38^. Conversely, Panavene et al. reported no serious adverse events following intraarticular administration of 6 mL of 16.7% DMSO into the knees of 17 patients with rheumatoid arthritis^39^. Murav'ev et al. also reported no serious adverse events following intraarticular administration of 3 mL of 20% DMSO into the knees of 17 patients with rheumatoid arthritis^40^. According to these studies, the intra-articular injection of DMSO at 5%, as used in the present study, should show no toxicity in human knee joints. Nevertheless, its repeated use in clinical practice should be carefully considered.

DMSO has also been reported to have an immunosuppressive effect, which may have affected the progression of OA in this study. We could not find any previous study that reported immunosuppressive effects of DMSO following its intraarticular injection in rats. Watson et al. examined the immunosuppressive effect of intraperitoneal administration of DMSO for 12 days on a rat collagen II autoimmune arthritis model. DMSO at 0.25 g/kg/day (3 g/kg) had no effect on arthritis, whereas at 5 g/kg/day (60 g/kg), DMSO reduced serum anti-collagen II IgG levels and delayed the onset of arthritis, but induced sterile peritonitis in all rats^41^. A toxicity study by Willson et al. also reported that intraperitoneal administration of DMSO at 0.5 g/kg caused peritonitis^42^. We injected 2.5 μL DMSO (assuming the specific gravity of DMSO to be 1.1 g/mL, this corresponds to 2.8 mg) into the knee (assuming a rat body weight of 200 g, this would be 14 mg/kg), which was about 1/4300 (= 14 mg / 60 g) of the amount of DMSO used by Watson et al. Therefore, the immunosuppressive effect of DMSO in this study is considered to be quite small.

Our study had three other limitations. One was our use of different fluids for the cultured MSCs and thawed MSCs for in vivo studies. Cultured MSCs were suspended in PBS and thawed MSCs were in 95% FBS with 5% DMSO. The effects of these different compositions on the experimental results are not known. For a more strict comparison, the same vehicle should have been used. A second limitation is the use of FBS instead of autologous rat serum for rat MSCs. For clinical applications, autologous human serum should be used as the serum component for safety reasons^43,44^. Although in vitro experiments were possible, experiments involving cell transplantation would have required a considerable amount of effort, which was not practicable. Another option would have been to use rat allogeneic serum instead of rat autologous serum. However, since FBS is more commonly used than rat allogeneic serum in experiments using rat cells^45,46^, FBS was used in the present study. A third limitation is that we did not examine the OA-inhibitory effect of thawed MSCs cryopreserved for 16 months. Clinically, the problem with thawed MSCs can be avoided by maintaining a relatively short cryopreservation period. Cryopreservation for 16 months decreased MSC viability by 8% and cartilage pellet formation by 22% by weight when compared to cultured MSCs. Clinically, this problem involving thawed MSCs could be avoided by limiting the cryopreservation to a relatively short period.

In conclusion, thawed cryopreserved MSCs and cultured MSCs showed a comparable inhibitory effect on OA progression in a rat meniscectomized OA model.

## Methods

### Rat synovial MSCs

All animal care and experiments were conducted in accordance with the ARRIVE guidelines and the institutional guidelines of the Animal Committee of Tokyo Medical and Dental University. Wildtype female Lewis rats (10 weeks of age, weighing 180–200 g) were purchased from Sankyo Labo Service Corporation, Inc. (Tokyo, Japan) (n = 40). Luciferase-expressing transgenic rats^47^ were also used for in vitro and vivo imaging (n = 4). The rats were kept in an environmentally controlled animal facility under a 12 h light/dark cycle with food and water ad libitum. Synovium was harvested from the rats’ infrapatellar fat pads. The synovium was minced and digested with collagenase (Merck, St. Louis, MO, USA) for 3 h. Synovial nucleated cells were cultured for 7–10 days in α-minimum essential medium (α-MEM; Thermo Fisher Scientific, Carlsbad, CA, USA), 10% fetal bovine serum (FBS; Thermo Fisher Scientific), and 1% antibiotic–antimycotic (Thermo Fisher Scientific) at 37 °C under 5% CO_2_. The resulting culture was collected and used as synovial MSCs^20,46,48^.

### Preparation of cultured and thawed cryopreserved MSCs

Cells at passage 3–4 were cultured for 1 week and then trypsinized. The cultured MSCs were prepared by resuspending in PBS at 1 × 10^6^ cells/mL for in vitro studies or 20 × 10^6^ cells/mL for transplantation. The cryopreserved MSCs were prepared by resuspending the cells containing 95% FBS and 5% dimethyl sulfoxide (DMSO; Fujifilm Wako, Tokyo, Japan)^22^ at 1 × 10^6^ cells/mL for in vitro studies or 20 × 10^6^ cells/mL for transplantation. Then, the MSCs were transferred to a bio freezing vessel (Bicell, Japan Freezer, Tokyo, Japan), placed in a freezer at − 80 °C overnight, and storing at − 150 °C for 6 days or 16 months. The tubes were removed from the freezing vessel and the frozen cells were thawed using a cell-thawing device (ThawSTAR, Astero Bio, Menlo Park CA, USA). As a negative control for in vitro studies, the cells were also frozen in 100% FBS. The composition of the cryopreservation solution (including the DMSO concentration) used in this study was based on our previous studies on human MSCs^22^.

### Viability

Cell viability was assessed using the trypan blue exclusion test and calculated by dividing the total number of live cells counted post-thaw by the number of cells originally frozen in the tube. Cellular dehydrogenase activity was tested by reacting the cells with the working solution for 30 min at 37 °C and quantified by a WST-8 assay (Dojindo, Kumamoto, Japan). The cell supernatant before and after preservation was also reacted at room temperature and assayed for lactate dehydrogenase (LDH) activity (Dojindo) by measuring the absorbance with a plate reader (Infinite M200; Tecan, Männedorf, Switzerland).

### Colony formation assay

A 0.5 μL volume of cell suspension (containing 500 cells, including living and dead cells) was plated in twelve 60 cm^2^ dishes and the cells were cultured for 14 days. Six dishes (dishes A, B, C, D, E, and F) were stained with crystal violet to count the total numbers of cell colonies. Colonies less than 2 mm in diameter were ignored^22,49^. The cells were harvested from the other 6 dishes (dishes G, H, I, J, K, and L) to count the cell numbers per dish with a hemocytometer. The cell number per colony was calculated based on the cell number from dish A divided by the colony number from dish G, and this calculation was repeated for the remaining pairs of dishes (i.e., B and H, C and I, D and J, E and K, and F and L). The mean and standard deviation were then determined for cell number per colony^22,49^.

### Chondrogenesis

A 250 μL volume of cell suspension (containing 2.5 × 10^5^ cells, including both living and dead cells) was added to six 15 mL tubes (Falcon) containing a chondrogenic induction medium consisting of Dulbecco's Modified Eagle Medium (DMEM; Thermo Fisher Scientific), 10 ng/mL transforming growth factor-β3 (TGF-β3, Miltenyi Biotec, Bergisch Gladbach, Germany), 500 ng/mL bone morphogenetic protein 2 (BMP-2, Medtronic, Minneapolis, MN, USA), 40 μg/mL proline (Merck), 100 nM dexamethasone, 100 μg/mL pyruvate (Merck), 1% antibiotic–antimycotic, 50 μg/mL ascorbate-2-phosphate, and 1% ITS + Premix (Becton Dickinson, San Jose, CA, USA). The cells were centrifuged at 450×*g* for 10 min to form cell pellets, which were cultured for 21 days. The cultured cell pellets were photographed and weighed with a semi micro balance (CPA225D, Sartorius, Gottingen, Germany). The pellets were cut into 5 µm sections and stained with safranin O and toluidine blue.

### Flow cytometry

Rat synovial MSCs at passage 3 were detached by treatment with TrypLE (Thermo Fisher Scientific) for 10 min and used for surface marker analysis. The cell fluorescence and percentage of antigen-positive cells were evaluated with a FACSVerse instrument (BD Biosciences). CD90-PE-Cy7 (eBioscience, San Diego, CA, USA), CD44-PE (eBioscience), CD105-APC (Novus Biologicals, Littleton, CO, USA), CD45-FITC (BD Pharmigen, San Jose, CA, USA) and CD34-PerCP-Cy5.5 (Novus Biologicals) antibodies were used. Cells were incubated with conjugated antibodies at 4 °C for 1 h in the dark. Cells positively stained with Ghost Dye Violet 510 (Tonbo Biosciences, San Diego, CA, USA) were removed as dead cells. Isotype controls were prepared as negative controls.

### Rat meniscectomized OA model

Rats were anesthetized by isoflurane inhalation. Both the right and left knee joints received surgery. After a medial parapatellar incision and lateral dislocation of the patellar tendon, the medial meniscus was exposed. The anterior insertional ligament of the medial meniscus was transected to dislocate the medial meniscus anteriorly, and the medial meniscus was resected at the level of the medial collateral ligament^27,47^. The wound was closed in layers. After the surgery, the rats were allowed to walk freely in their cages.

### Bioluminescence imaging

Synovial MSCs derived from luciferase-expressing transgenic rats (Luc^+^ MSCs) were used. Cultured and thawed MSCs were plated into 96-well plate at the density of 10^3^, 10^4^, 10^5^, and 10^6^ cells/well. d-Luciferin potassium salt (200 μg/mL, ab143655, abcam, Cambridge, UK) was added to each well and the luminescence intensity was quantified using IVIS Lumina XRMS series III instrument (SPI, Tokyo, Japan). A sample containing 1 × 10^6^ cultured and thawed Luc^+^ MSCs were injected two months after the surgery. D-luciferin (20 mg/mL, 50 μL) was injected into the knees at 1, 4, and 7 days after the transplantation, and photons were detected with the IVIS instrument (n = 4). The luminescence intensity was quantified as photon flux in units of photons per seconds in the region of each knee.

### Validation of the inhibitory effect of cultured and thawed MSCs on OA progression

A 50 μL volume of PBS was injected into one knee (PBS group) and 1 × 10^6^ cultured MSCs at passage 3 suspended in 50 μL of PBS were injected into contralateral knee (Cultured group) every week beginning 2 weeks after the surgery (n = 12). In another group of rats, 50 μL of 95% FBS with 5% DMSO was injected into one knee (95%FBS group) and 1 × 10^6^ thawed MSCs at passage 3 (suspended in 50 μL of 95% FBS with 5% DMSO with no compensation for viability) were injected into contralateral knee (Thawed group) every week beginning 2 weeks after the surgery (n = 12).

### Direct comparison between cultured MSCs and thawed MSC

We injected 1 × 10^6^ cultured MSCs at passage 3 suspended in PBS into the left and right sides of one knee (Cultured group) and 1 × 10^6^ thawed MSCs at passage 3 (suspended in 95% FBS with 5% DMSO with no compensation for viability) into the sides of the contralateral knee (Thawed group) every week beginning 2 weeks after the surgery. All knees were evaluated at 8 weeks (n = 9).

### Evaluations of cartilage degeneration

Both the tibial and femoral condyles were removed separately and evaluated by India ink staining for macroscopic observation. Histological examinations were conducted by fixing both tibial and femoral cartilage in 10% formalin neutral buffer solution for 2 days and decalcifying with 20% ethylenediaminetetraacetic acid (EDTA; Fujifilm Wako, Tokyo, Japan) for 2 weeks, followed by paraffin wax embedding. The specimens of both medial condyles were sectioned in the sagittal plane at 5 µm and stained with safranin O and fast green. Cartilage degeneration was evaluated using a modified “gross finding score” (Supplementary Table 1)^27^ and the Osteoarthritis Research Society International (OARSI) scoring system for histology^50^. Three different researchers independently scored the sections in a blinded manner and the median value was selected.

### Statistical analysis

Statistical analysis was performed with the Graph-Pad Prism 6 (GraphPad Software, CA, USA). For multiple comparisons, data were analyzed using repeated measures one-way ANOVA followed by Tukey’s multiple comparisons or Kruskal–Wallis test followed by Dunn’s multiple comparisons. Comparisons between the unpaired groups were analyzed using the Student’s t test. Comparisons between the matched pairs were analyzed using the Wilcoxon's signed rank test. A P value of < 0.05 was considered statistically significant. Before comparing the cultured and thawed groups directly, we calculated the sample size with a significance level of 5% and a power of 80% based on the OARSI histological score in tibial cartilage between the PBS-injected and the cultured MSC-injected knees. Data were expressed as mean ± standard deviation. The methods of statistical analysis are described in each of the figure legends.

### Ethics approval

All experimental protocols and studies were approved by animal care and use committee (reference number: A2019-197C) and genetically modified organisms safety committee (reference number: G2019-019C) of Tokyo Medical and Dental University. All animal care and experiments were performed in accordance with the institutional guidelines of the Animal Committee of Tokyo Medical and Dental University.

## Supplementary Information

Supplementary Information 1.

Supplementary Information 2.

Supplementary Information 3.

Supplementary Information 4.

Supplementary Information 5.

## Data Availability

The datasets obtained and analyzed in the current study are available from the corresponding author on reasonable request.
