# Supplementary material for: Thawed cryopreserved synovial mesenchymal stem cells show comparable effects to cultured cells in the inhibition of osteoarthritis progression in rats
Source: Sci Rep. 2021 May 6;11:9683. doi: 10.1038/s41598-021-89239-8 (PMC8102597; doi:10.1038/s41598-021-89239-8)
Supplement: Supplementary file 1 — Supplementary Information 1. [file 41598_2021_89239_MOESM1_ESM.pdf]

Thawed cryopreserved synovial mesenchymal stem cells show comparable effects to cultured cells in the inhibition of osteoarthritis progression in rats

Kiyotaka Horiuchi[1], Nobutake Ozeki[1], Kentaro Endo[1], Mitsuru Mizuno[1], Hisako Katano[1], Masako Akiyama[2], Kunikazu Tsuji[3], Hideyuki Koga[4] and Ichiro Sekiya[1]

[1]Center for Stem Cell and Regenerative Medicine, Tokyo Medical and Dental University, 1-5-45, Bunkyo-ku, Yushima, Tokyo, Japan

[2]Research Administration Division, Tokyo Medical and Dental University, Tokyo, Japan.

[3]Department of Cartilage Regeneration, Tokyo Medical and Dental University, Tokyo, Japan.

[4]Department of Joint Surgery and Sports Medicine, Tokyo Medical and Dental University, Tokyo, Japan.

*** Correspondence information:**
Ichiro Sekiya, MD, PhD
Director and Professor, Center for Stem Cell and Regenerative Medicine
Tokyo Medical and Dental University
1-5-45 Yushima, Bunkyo-ku, Tokyo 113-8510, Japan
Phone: +81-3-5803-4017
FAX: +81-3-5803-0192
E-mail: sekiya.arm@tmd.ac.jp

SUPPLEMENTARY INFORMATION

Additional supporting information may be found in the online version of this article:

Supplementary Figure 1

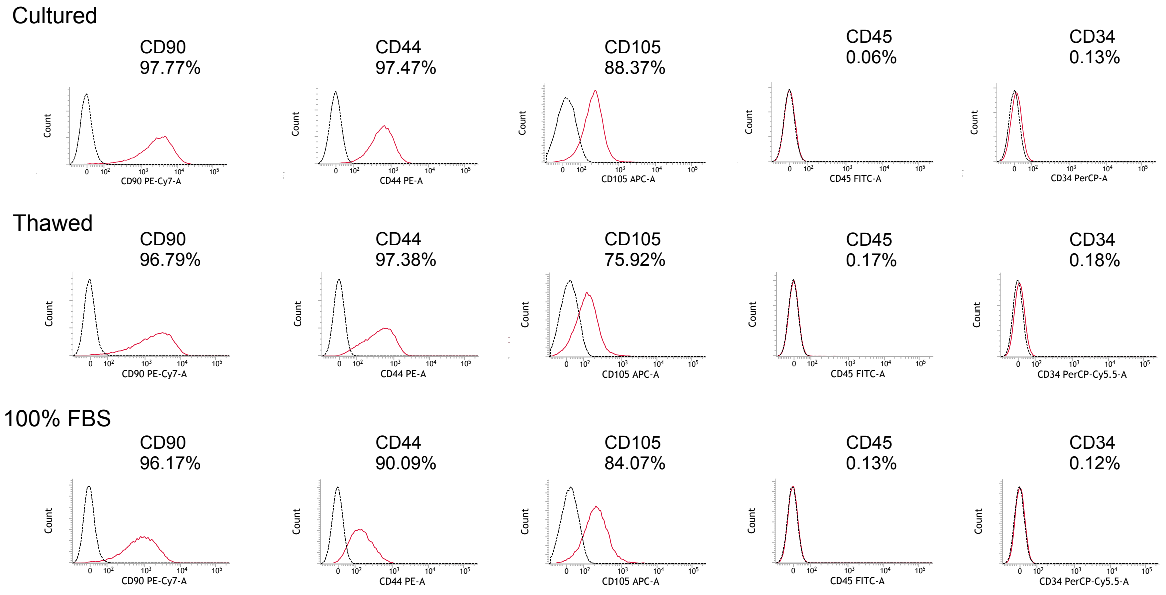

<u>**Supplementary Figure 1. Representative histogram of surface epitopes.** Black line shows isotype control.</u>
