# Supplementary material for: Thawed cryopreserved synovial mesenchymal stem cells show comparable effects to cultured cells in the inhibition of osteoarthritis progression in rats
Source: Sci Rep. 2021 May 6;11:9683. doi: 10.1038/s41598-021-89239-8 (PMC8102597; doi:10.1038/s41598-021-89239-8)
Supplement: Supplementary file 2 — Supplementary Information 2. [file 41598_2021_89239_MOESM2_ESM.pdf]

Thawed cryopreserved synovial mesenchymal stem cells show comparable effects to cultured cells in the inhibition of osteoarthritis progression in rats

Kiyotaka Horiuchi[1], Nobutake Ozeki[1], Kentaro Endo[1], Mitsuru Mizuno[1], Hisako Katano[1], Masako Akiyama[2], Kunikazu Tsuji[3], Hideyuki Koga[4] and Ichiro Sekiya[1]

[1]Center for Stem Cell and Regenerative Medicine, Tokyo Medical and Dental University, 1-5-45, Bunkyo-ku, Yushima, Tokyo, Japan

[2]Research Administration Division, Tokyo Medical and Dental University, Tokyo, Japan.

[3]Department of Cartilage Regeneration, Tokyo Medical and Dental University, Tokyo, Japan.

[4]Department of Joint Surgery and Sports Medicine, Tokyo Medical and Dental University, Tokyo, Japan.

*** Correspondence information:**
Ichiro Sekiya, MD, PhD
Director and Professor, Center for Stem Cell and Regenerative Medicine
Tokyo Medical and Dental University
1-5-45 Yushima, Bunkyo-ku, Tokyo 113-8510, Japan
Phone: +81-3-5803-4017
FAX: +81-3-5803-0192
E-mail: sekiya.arm@tmd.ac.jp

SUPPLEMENTARY INFORMATION

Additional supporting information may be found in the online version of this article:

Supplementary Figure 2

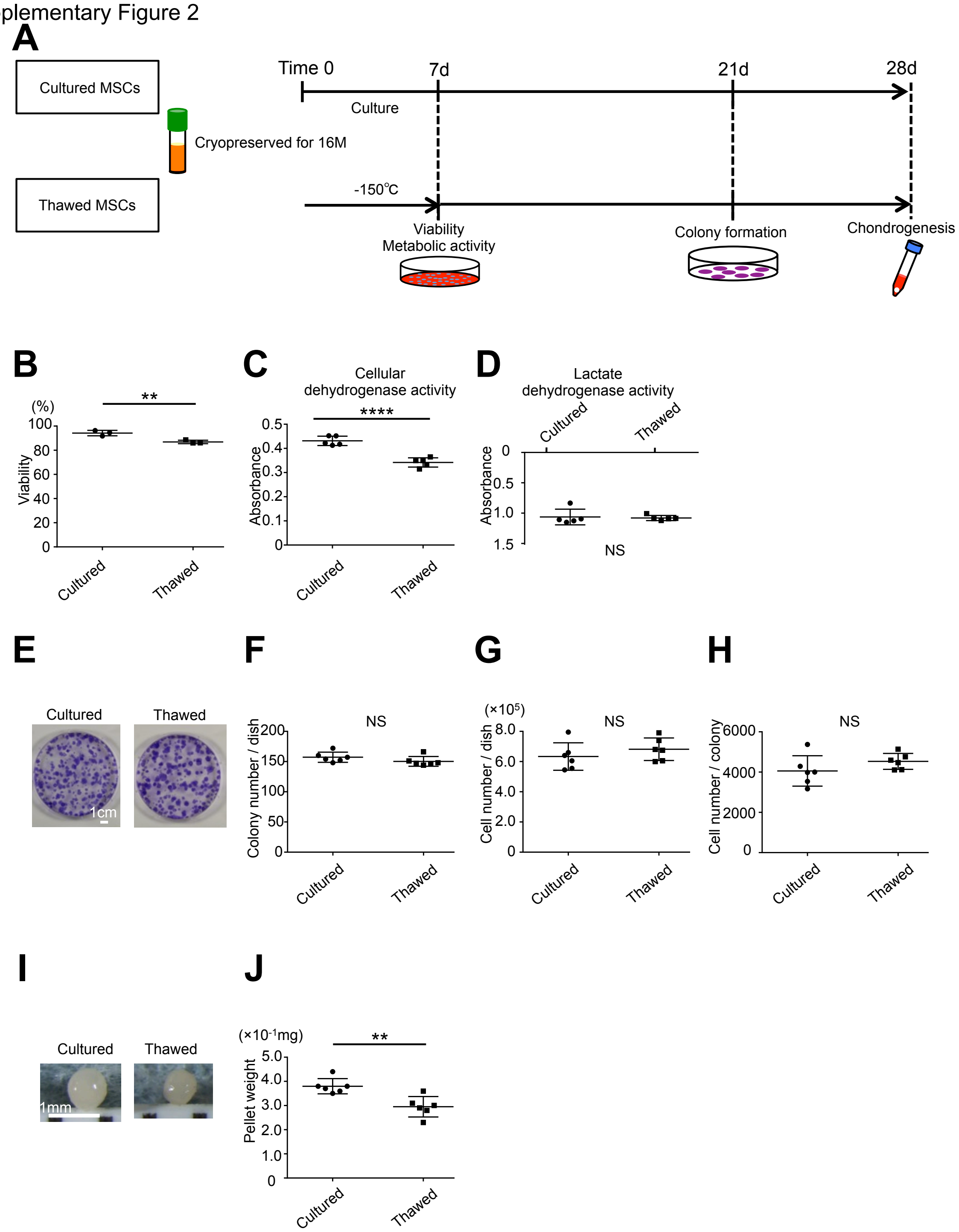

Supplementary Figure 2. Effects of 16 months of cryopreservation on the in vitro viability and properties of rat synovial MSCs. (A) Scheme: A sample of 1×10^6 synovial MSCs suspended in 1000 μL preservation fluid containing 95% FBS with 5% DMSO was cryopreserved at −150 °C for 16 months and then used as thawed MSCs. Thawed MSCs were also cultured for 1 week for use as cultured MSCs. The cells were analyzed for viability and metabolic activity. A 0.5 μL volume of cell suspension (containing 500 cells, including living and dead cells) was allocated to a 60 cm^2 dish and cultured for colony formation. A 250 μL volume of cell suspension (containing 2.5×10^5 cells, including living and dead cells) was allocated to a 15 mL tube and cultured for chondrogenesis. (B) Viability was assessed by trypan blue staining. The average with SD is shown (n=3). (C) Cellular dehydrogenase activity was used to confirm live cell metabolic activity (n=5). (D) Lactate dehydrogenase activity was used as a marker of dead cells (n=5). (E) Colony formation: colonies were stained with crystal violet. (F) Colony number per dish (n=6). (G) Cell number per dish (n=6). (H) Cell number per colony (n=6). (I) Cartilage pellets. (J) Cartilage pellet weight (n=6). ND, not detected; NS, not significant; **$p < 0.01$, ****$p < 0.0001$ by Student's t-test (Fig. 1B, C and D) or Mann-Whitney's U test (Fig. 1F, G, H and J).
