# Supplementary material for: Thawed cryopreserved synovial mesenchymal stem cells show comparable effects to cultured cells in the inhibition of osteoarthritis progression in rats
Source: Sci Rep. 2021 May 6;11:9683. doi: 10.1038/s41598-021-89239-8 (PMC8102597; doi:10.1038/s41598-021-89239-8)
Supplement: Supplementary file 3 — Supplementary Information 3. [file 41598_2021_89239_MOESM3_ESM.pdf]

Thawed cryopreserved synovial mesenchymal stem cells show comparable effects to cultured cells in the inhibition of osteoarthritis progression in rats

Kiyotaka Horiuchi[1], Nobutake Ozeki[1], Kentaro Endo[1], Mitsuru Mizuno[1], Hisako Katano[1], Masako Akiyama[2], Kunikazu Tsuji[3], Hideyuki Koga[4] and Ichiro Sekiya[1]

[1]Center for Stem Cell and Regenerative Medicine, Tokyo Medical and Dental University, 1-5-45, Bunkyo-ku, Yushima, Tokyo, Japan

[2]Research Administration Division, Tokyo Medical and Dental University, Tokyo, Japan.

[3]Department of Cartilage Regeneration, Tokyo Medical and Dental University, Tokyo, Japan.

[4]Department of Joint Surgery and Sports Medicine, Tokyo Medical and Dental University, Tokyo, Japan.

*** Correspondence information:**
Ichiro Sekiya, MD, PhD
Director and Professor, Center for Stem Cell and Regenerative Medicine
Tokyo Medical and Dental University
1-5-45 Yushima, Bunkyo-ku, Tokyo 113-8510, Japan
Phone: +81-3-5803-4017
FAX: +81-3-5803-0192
E-mail: sekiya.arm@tmd.ac.jp

SUPPLEMENTARY INFORMATION

Additional supporting information may be found in the online version of this article:

Supplementary Figure 3

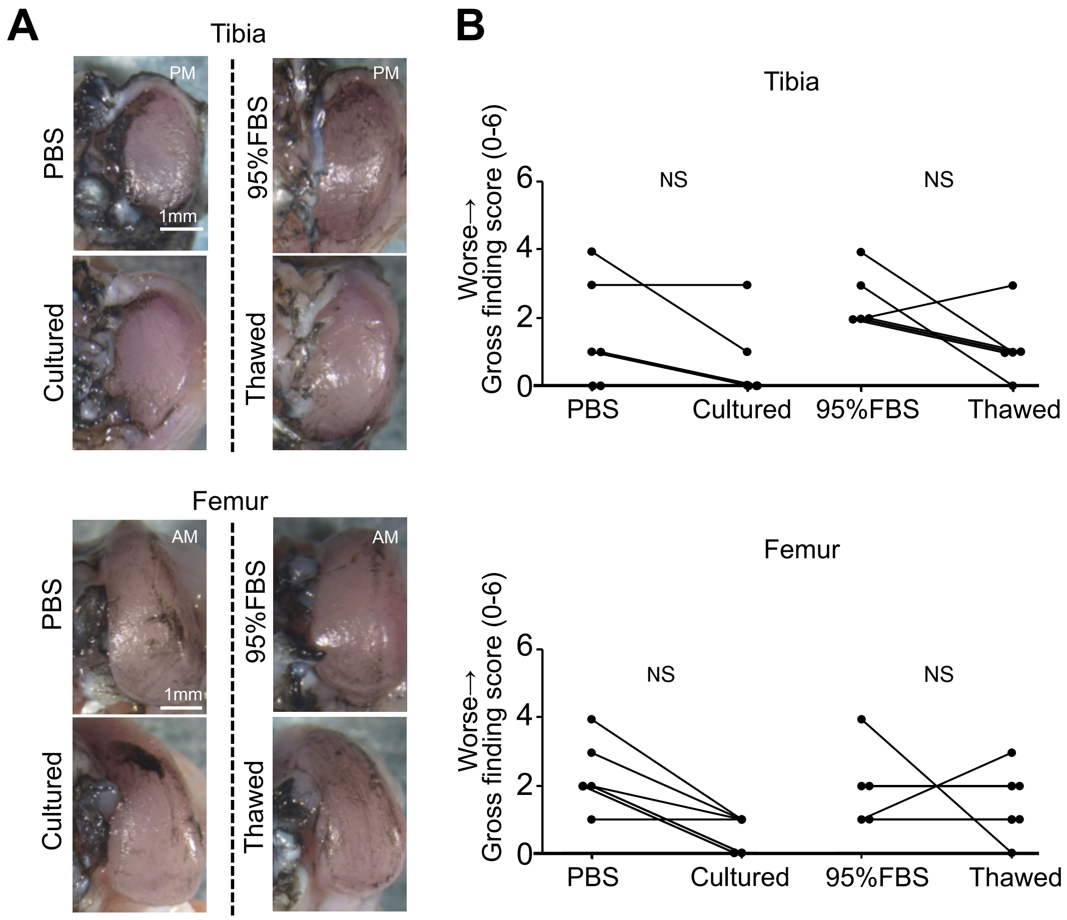

Supplementary Figure 3. Macroscopic analysis of cartilage four weeks after injections of cultured MSCs versus thawed cryopreserved MSCs in a rat OA model. The knee cartilage was assessed to compare the left and right sides of the same individual. (A) Representative macroscopic images for medial tibial and femoral condyles stained with India ink. The images in the cultured and the thawed groups are inverted horizontally for ease of comparison. PM, posteromedial; AM, anteromedial. (B) Gross finding score. NS, not significant by Wilcoxon's signed rank test (n=6).
