# Supplementary material for: Thawed cryopreserved synovial mesenchymal stem cells show comparable effects to cultured cells in the inhibition of osteoarthritis progression in rats
Source: Sci Rep. 2021 May 6;11:9683. doi: 10.1038/s41598-021-89239-8 (PMC8102597; doi:10.1038/s41598-021-89239-8)
Supplement: Supplementary file 5 — Supplementary Information 5. [file 41598_2021_89239_MOESM5_ESM.docx]

**Thawed cryopreserved synovial mesenchymal stem cells show comparable effects to cultured cells in the inhibition of osteoarthritis progression in rats**

Kiyotaka Horiuchi^1^, Nobutake Ozeki^1^, Kentaro Endo^1^, Mitsuru Mizuno^1^, Hisako Katano^1^,

Masako Akiyama^2^, Kunikazu Tsuji^3^, Hideyuki Koga^4^ and Ichiro Sekiya^1^

^1^Center for Stem Cell and Regenerative Medicine, Tokyo Medical and Dental University, 1-5-45, Bunkyo-ku, Yushima, Tokyo, Japan

^2^Research Administration Division, Tokyo Medical and Dental University, Tokyo, Japan.

^3^Department of Cartilage Regeneration, Tokyo Medical and Dental University, Tokyo, Japan.

^4^Department of Joint Surgery and Sports Medicine, Tokyo Medical and Dental University, Tokyo, Japan.

* **Correspondence information:**

Ichiro Sekiya, MD, PhD

Director and Professor, Center for Stem Cell and Regenerative Medicine

Tokyo Medical and Dental University

1-5-45 Yushima, Bunkyo-ku, Tokyo 113-8510, Japan

Phone: +81-3-5803-4017

FAX: +81-3-5803-0192

E-mail: [sekiya.arm@tmd.ac.jp](mailto:sekiya.arm@tmd.ac.jp)

**SUPPLEMENTARY INFORMATION**

Additional supporting information may be found in the online version of this article:

**Table 1**

**Gross finding score: macroscopic cartilage scoring (0–6)**

Grade Findings

0 Intact articular surface

1 Fibrillation (<0.5mm)

2 Fibrillation (≥0.5mm)

3 Width of erosion area (<0.5mm)

4 Width of erosion area (≥0.5mm, <1mm)

5 Width of erosion area (≥1mm, <1.5mm)

6 Width of erosion area (≥1.5mm, <2mm)

Both the medial tibial and femoral condyle were evaluated separately, and the higher point value was selected.
